# Effect of F0 contour on perception of Mandarin Chinese speech against masking

**DOI:** 10.1371/journal.pone.0209976

**Published:** 2019-01-03

**Authors:** Meihong Wu

**Affiliations:** School of Information Science and Engineering, Xiamen University, Fujian, China; University College London, UNITED KINGDOM

## Abstract

Intonation has many perceptually significant functions in language that contribute to speech recognition. This study aims to investigate whether intonation cues affect the unmasking of Mandarin Chinese speech in the presence of interfering sounds. Specifically, intelligibility of multi-tone Mandarin Chinese sentences with maskers consisting of either two-talker speech or steady-state noise was measured in three (flattened, typical, and exaggerated) intonation conditions. Different from most of the previous studies, the present study only manipulate and modify the intonation information but preserve tone information. The results showed that recognition of the final keywords in multi-tone Mandarin Chinese sentences was much better under the original F0 contour condition than the decreased F0 contour or exaggerated F0 contour conditions whenever there was a noise or speech masker, and an exaggerated F0 contour reduced the intelligibility of Mandarin Chinese more under the speech masker condition than that under the noise masker condition. These results suggested that speech in a tone language (Mandarin Chinese) is harder to understand when the intonation is unnatural, even if the tone information is preserved, and an unnatural intonation contour decreases releasing Mandarin Chinese speech from masking, especially in a multi-person talking environment.

## Introduction

Speech recognition and auditory comprehension are some of the most important activities encountered in everyday life. In everyday communication situations, human listeners rely on a variety of perceptual strategies to segregate the attended target speech from background competing sounds. Intonation has many perceptually significant functions in language that contribute to the comprehension and recognition of speech[1,2]. Specifically, intonation cues are important for conveying the intended meaning of clusters of discourse[3] and deliver information at levels higher than the word.

The literature has investigated the role of intonation in speech intelligibility by flattening the fundamental frequency (F0) contours of normal speech and comparing the recognition of these stimuli to naturally intonated speech[4]. The findings within these studies have indicated a detrimental effect of flattening the F0 contour on sentence intelligibility. Among the explanations proposed for these findings was that the rise and fall of the F0 contour has been found to direct the listener's attention to the content words of the utterance; therefore, without these cues, the intelligibility of the utterance decreases[5].

Binns and Culling (2007)[5] also assessed the role intonation contours play in speech intelligibility by manipulating the F0 contours. The manipulations involved either reducing the F0 incrementally from the variation present in a normally intonated contour to a flattened contour (where the variation has been removed), with no variation, or a complete inversion of the F0 contour. They found that naturally varying F0 contours improved speech intelligibility in background noise compared with flat or inverted F0 contours, and their results also indicated that reducing the amount of F0 variation has a progressively detrimental effect on speech intelligibility. The authors suggested that in the flattened F0 condition, the words all had the same F0 and lacked the important stress-based segmentation cue [6], thus, the stress cues based on F0 were simply neutralized across the individual words in the sentences as opposed to being inaccurate or misleading. Therefore, in one argumentation, the speech utterance with the absence of F0 variation has lower intelligibility than speech with normal F0 variation.

Binns and Culling (2007) discussed that where there is an F0 contour in the right direction, the focused words may remain focused, to some degree, and the surrounding contour still guides the listener toward those words more readily than in a flattened F0 condition. Their results showed that even a small amount of F0 modulation (m = 0.25) increased the intelligibility of the sentences against a single-talker interferer, indicating it is the contour shape that is important to speech recognition. Moreover, the literature has demonstrated that humans with hearing impairment require greater F0 variation in speech to follow intonation patterns compared with humans with normal hearing[7].

The aforementioned results of these studies led to one of the hypotheses in this study: an exaggerated F0 contour may improve performance relative to a decreased F0 variation condition. In addition, if a greater moment-to-moment variation in the F0 contour exists, the intelligibility of the speech utterance increases; according to our review of the literature, this phenomenon has not been well documented. In other words, whether the greater F0 variation in the contour may have greater benefits for speech intelligibility is still not clear and requires further analysis. Considering this information, we questioned whether exaggerating the F0 contour to some degree could affect the speech intelligibility. Thus, one of the purposes of this study was to investigate whether an exaggerated F0 affects the speech intelligibility relative to the naturally produced speech utterance as much as a reduction of F0 modulation.

Furthermore, a noise background was used in Binns and Culling (2007), and the speech recognition thresholds were measured in interfering speech or in speech-shaped noise. Binns and Culling (2007) observed that flattened speech degraded speech intelligibility relative to typically intonated speech, and this difference did not reach significance when using a speech-shaped background noise masker. However, the authors did find a significant difference in speech intelligibility between the two conditions when the masker’s interfering speech had a variety of F0 contours (e.g., flattened F0, normal F0, and inverse F0). In Laures and Bunton (2003), the unmodified and flattened F0 utterances masked by non-speech (white noise) or speech-like (multi-speaker babble) noise was examined to obtain the measures of speech intelligibility. Most notably, their results found that utterances with a flattened F0 contour were less intelligible than utterances with a varying F0 in noisy conditions, regardless of the type of noise[8]. Hence, in terms of interferers, the effect of intonation on speech intelligibility under adverse listening conditions still requires further investigation.

Brungart et al. (2001) showed that using the same talker for the target and the interferer generated more masking than using a different talker of the same sex, which created more of a masking effect than using a talker of the opposite sex. The proposed cause of this effect is the increase in informational masking, because the number of voices is initially increased to two voices; then, a consequent decrease as the number of voices increases to three [9]. Thus, the two-talker babble has a much greater negative effect than the multi-talker babble. Therefore, according to the results in Brungart et al. (2001), in this study, the masker was speech-like (e.g., two-speaker babble) or a continuous nature (e.g., white noise) was used to simulate the effect on speech intelligibility in an adverse listening background.

Notably, most of the aforementioned studies have been conducted using English stimuli and English listeners. Few studies have been conducted on tonal languages. In a tonal language such as Mandarin Chinese, F0 serves as the primary acoustic cue for both lexical tones and sentence intonation, and the syllable varies in tones which make the contour of sentence intonation colourful and varied.

In a study with Mandarin Chinese stimuli, sentences with flattened F0 and normal F0 contours were used to investigate the importance of F0 variation for Mandarin speech comprehension; notably, the results showed that sentences with flattened F0 contours were as intelligible as those with normal F0 patterns in a quiet environment[10]. Wang et al. (2013)[11] indicated that the speech materials used in Patel et al., (2010)[10] were semantically predictable meaningful sentences, and because the top-down information of the sentence context would provide a crucial role in guiding speech perception[12], what contributed to the unchanged intelligibility of monotonous Mandarin sentences with flattened F0 contours in quiet was unclear[11]. Wang et al. (2013) further evaluated speech intelligibility of the Mandarin sentences and word lists with or without F0 variations, and the results showed that listeners can automatically use additional neural and cognitive resources to recover disrupted F0 contour patterns in sentences. Particularly, significant interactions existed among the three factors (i.e., sentence context, F0 variation, and background noise) in the intelligibility of Mandarin speech.

Since there was evidence that the semantic information provided by sentence context contributes to speech intelligibility in Wang et al. (2013), in this study, Chinese “nonsense” sentences[13,14], which were syntactically correct but not semantically meaningful, was used in this study to ensure that the listeners can't make relatively use of contextual cues to predict the keywords in the sentences.

In summary, the present study were intended to clarify whether the tone information for Mandarin Chinese was preserved but the intonation information was manipulated (flattened or exaggerated) could affect the ability to understand speech in the presence of two types of interfering sounds, speech masker and noise masker. Specifically, speech recognition in noise was measured for Mandarin sentences having a typical F0 contour, a decreased F0 contour, or an exaggerated F0 contour, and the effects by decreasing the amount of F0 variation present in a standard contour of Mandarin Chinese sentences along with exaggerating the F0 contour was investigated.

## Materials and methods

### Participants

Twelve volunteers participated in this study (7 females and 5 males aged 20 to 26 years with a mean age of 24.0 years). The participants were recruited from the local community, and their first language was Mandarin Chinese. All the participants gave their written informed consent to participate in the study. The experimental procedures of the present study involving human subjects were approved by the ethics review committee in Xiamen University.

All the participants had symmetrical hearing (≤ 20 dB difference between the two ears). Pure-tone hearing thresholds were no greater than 25 dB HL between 0.125 and 8 kHz.

**Fig 1:**
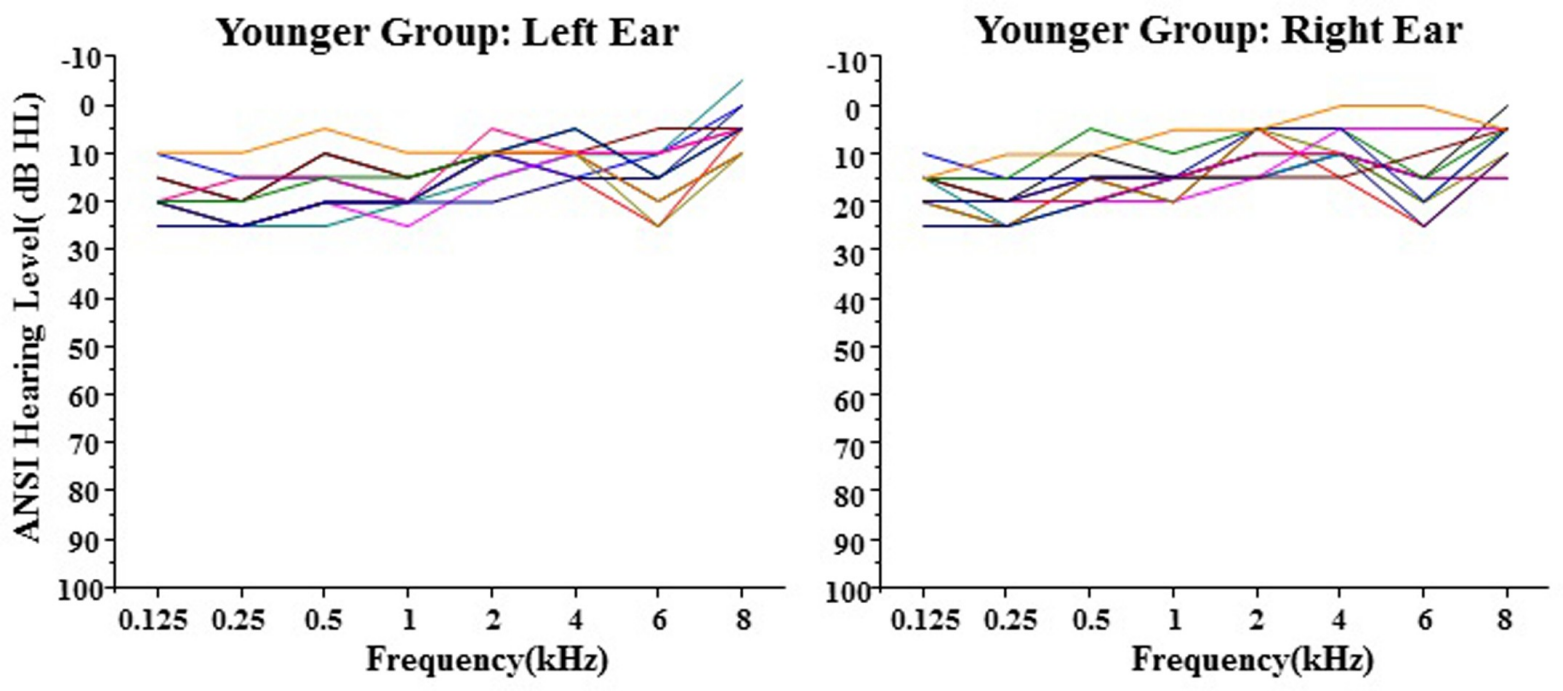
Average hearing thresholds in the left ear (filled circles) and right ear (open circles). ANSI: American National Standards Institute (S3.6–1989). Error bars represent the standard errors of the mean.

Fig 1 presents the group mean of the hearing thresholds as a function of frequency.

### Apparatus

An experiment was conducted in an anechoic chamber (Beijing CA Acoustics Co. Ltd, Beijing, China; 560 cm in length, 400 cm in width, and 193 cm in height). Acoustic signals were digitized at a sampling rate of 22.05 kHz with a 24-bit amplitude quantization. The acoustic signals were presented over a loudspeaker (Dynaudio Acoustics, BM6 A, Denmark) at 0o azimuth and at elevation relative to the participant. The loudspeaker height was 106 cm, which was almost ear level for a seated participant. The distance from the loudspeaker to the centre of the participant’s head was 185 cm.

### Stimuli

#### Multi-tone Chinese “nonsense” sentences

The speech materials consisted of sentences from a Chinese “nonsense” corpus generated by a speech and hearing research group at Peking University (see more details in Yang et.al, 2007).

Speech stimuli were Chinese “nonsense” sentences that were grammatically correct but not semantically meaningful [14]. All the sentences had a subject-predicate-object structure, and each sentence had 12 syllables, including three keywords with two syllables each. Here is an example of one Chinese nonsense sentence, the English translation of which is, “This cod has drift that notes” (keywords are underlined).

About 6000 high frequencies occurred disyllabic verbs and 12000 high frequencies occurred disyllabic nouns from the database published by People's Daily for more than 9 years were used to be combined randomly into 6000 Chinese “nonsense” sentences. The sentences with zero probability of co-occurrence of keywords in the database were selected and used in the present study for the text-to-speech transformation. To guarantee both high quality and uniformity of the acoustical features of these sentences stimuli, the speeches were generated by speech synthesis technique.

The target speech was recited by an artificially synthesized young, female voice. The generation of artificially synthesized target speech signal was briefly described as follows: First, a news-broadcast style Chinese corpus (Blizzard Challenge 2009) was sampled for model training, and then some critical speech-acoustical parameters (including the mel-cepstrum,log F0, and band aperiodicity measures and so on.) were extracted and the five-state left-to right Hidden Markov Model structure was adopted. Second, the speech-parameter sequence for each sentence stimulus was generated from the corresponding Hidden Markov Model, and then a speech waveform was synthesized using the algorithm of the Mel Log Spectrum Approximation Filter with the generated parameters, and finally, the initial acoustical model was established by a training procedure using the speech corpus with selected vocal characteristics.

Speech samples (about 432 sentences and lasting 40 min) were processed by the initial acoustic model as described above to obtain the acoustical model for each of the target voices with different intonation pattern(see details in the following section Synthesized intonation speech). Consequently, for each of the target voices, using the resultant target-voice acoustical models, written nonsense sentences were transformed into speech signals with the speaker’s vocal characteristics (including fundamental frequency, characteristic pitch glides, F0 contour).

#### Synthesized intonation speech

In the generation of an intonation pattern of a speech synthesis, a speech synthesis system is capable of providing highly natural speech and reproducing speech characteristics of a speaker flexibly and accurately by effectively utilizing fundamental frequency (F0) patterns of actual speech accumulated in a database[15].

In early modelling studies of Mandarin intonation, intonation was insensitive to lexical tones and determined before tone selection in most of these models. Recently, quantitative modelling of Chinese intonation in which the interaction between tone and intonation was quantified through simulating surface F0’s has been investigated [16,17,18].

In this study, a hierarchical pitch target model and a hierarchical duration model, which were proposed in Zhang et al. (2010), were used to capture the hierarchical characteristics of prosodic features. At first, a Mandarin speech corpus including 1,000 declarative utterances was selected from a classical database of Blizzard Challenge 2009(Blizzard Challenge 2009). Each stimuli utterance contained 15–60 syllables and was recited by a younger female speaker. Next, the parameters of the model were trained and learned from a Mandarin speech corpus, which reveal the forms of tones and characteristic pitch trend in each prosodic layer in fluent speech. The prosodic layers consisted of syllable, prosodic word, prosodic phrase, intonation phrase, and utterance. The pitch target has a hierarchical structure and was generated by adding up a tonal pattern from the syllable layer and biases from higher prosodic layers. The biases from a prosodic unit form a linear sequence along syllables in unit domain and acted as intonation attached to tones [19]. Finally, the current model parameters were used to synthesize pitch contours in the learning process. The pitch contour is viewed as a superposition of a tone curve and intonation curve[16]; thus, the two curves from the observed pitch contour can be separated by filtering, and the parameters related to the tone and intonation can be extracted from the separated curves, respectively. It is worth noting that different from most of the previous studies where both intonation and tone were all flattened in the resulting flattened monotonous sentence, the present study only manipulated and modified the intonation. After intonation contour modelling, the parameters were predicted, and the parametric model can approximate the intonation precisely[16,17,18].

This study adopted the aforementioned method to quantitatively model Mandarin Chinese intonation, in which the interaction between tone and intonation was quantified through simulating surface F0s and then developed different degrees of intonation cue.

**Fig 2:**
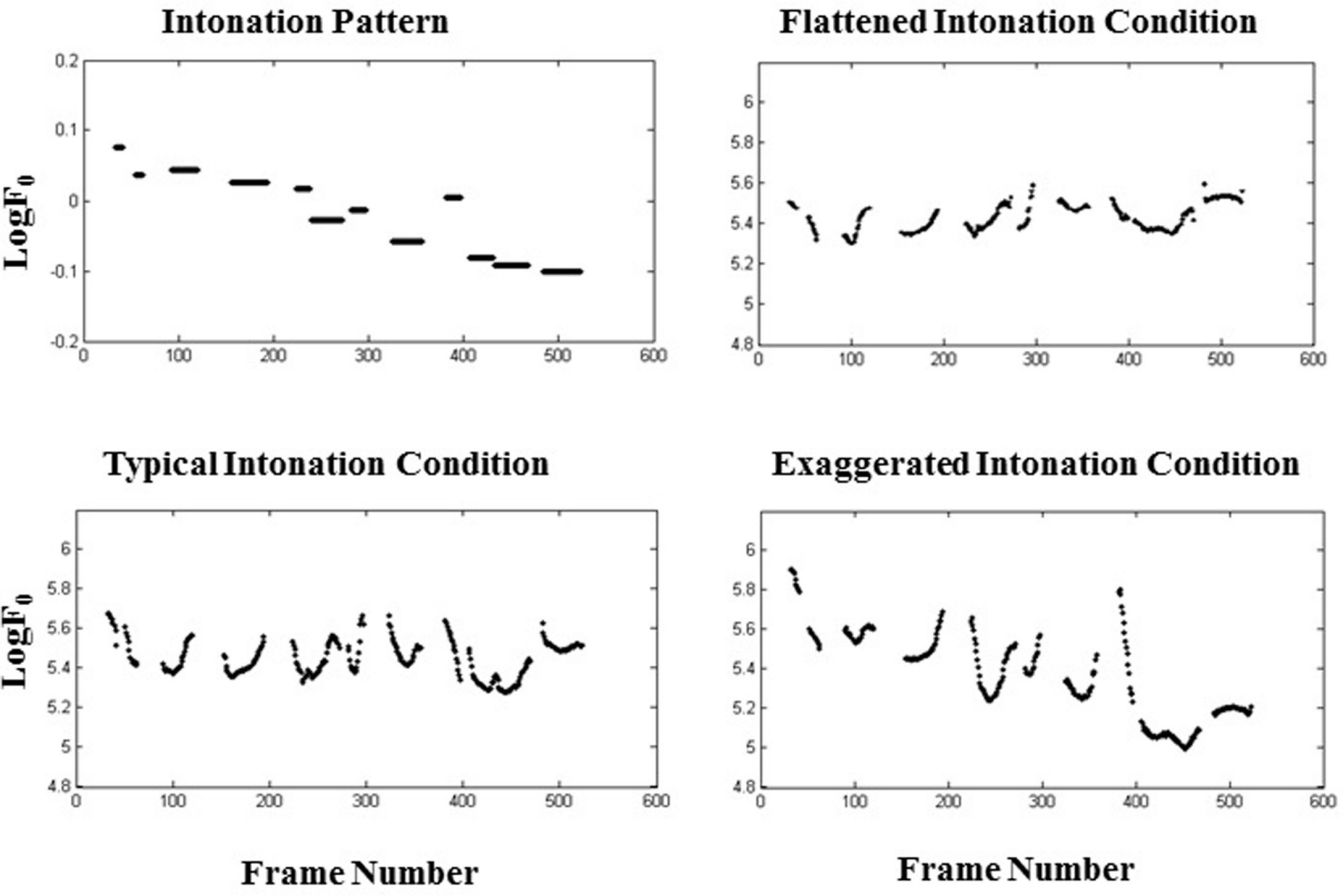
**Sketch map of an example of a target sentence with its intonation pattern (left top panel) and three types of intonation:** (1) flattened intonation conditions (top-right panel), (2) typical intonation conditions (bottom-left panel), and (3) exaggerated intonation conditions (bottom-right panel).

There are three intonation conditions in this study. Speech recognition in noise was examined for utterance stimuli following three types of intonation: flattened intonation, typical intonation, and exaggerated intonation (Fig 2).

1. Flattened intonation: resynthesized the “nonsense” Chinese sentences with a monotonous intonation contour by compressing the value of dynamic range of intonation biases to the observed original pitch target into 0.5 (i.e. compressing the magnitude of F0).
2. Typical intonation: unmodified, natural normal intonation contour of “nonsense” Chinese sentences (i.e. original F0).
3. Exaggerated intonation: resynthesized the experimental sentences with a normal intonation contour by exaggerating 4 times the value of dynamic range of intonation biases to the separated original intonation curve (i.e. expanding the magnitude of F0).

In general, the typical intonation was similar to the usually natural familiar intonation, flattened intonation was a natural but "decreased" intonation, and exaggerated intonation was a natural but "exaggerated" intonation.

The standard contour referred to the normal intonation placed on that sentence. The flattened intonation condition followed the same general shape of the standard contour, but the amount of variation in both is greatly reduced, and the exaggerated intonation condition referred to an amplified F0 contour in which the variation is 4 times that of the standard contour.

Speech samples (approximately 900 sentences and a duration of 60 min) of each of the three intonation phrases (flattened intonation; typical intonation; exaggerated intonation) were added into the initial acoustic model to obtain the acoustical model for each of the three intonations through the model adaptive procedure.

Six native Mandarin Chinese speaking younger adults (6 females ages between 23 and 26 years with a mean age of 24.6) were recruited to evaluate the quality of the artificially synthesized voices. Eight younger Chinese speaking listeners (6 females and 2 males) with the mean age of 24 were invited for evaluating the naturalness of the speech. All these participants gave their written informed consent to participate in this testing, and this experimental procedures was part of the protocol approved by the ethics review committee in Xiamen University. They were presented sentences recited separately by three intonation voices, that is, an artificial flattened intonation, typical intonation, and exaggerated intonation by young female. The listeners were informed about the number of intonation types and asked to repeat each of the speech presentations.

The results showed that the accuracy for recognizing the keywords was reaching up to 99% in younger adults under the quiet condition and under the flattened, typical, and exaggerated intonation conditions across the keyword positions. To some extent, these results are consistent with those in [20], which evaluated the recognition of sine-wave Mandarin sentences and showed that nearly perfect sentence recognition was obtained despite the chance level recognition of lexical tones. Moreover, in agreement with previous findings on some level, this result contributes to the speculation in[21], that lexical tones are relatively redundant cues for Mandarin sentence intelligibility when no masker is presented, and other cues could compensate for the distorted lexical tone contour.

The naturalness of speech is rated in terms of the subjectively evaluated method Mean Opinion Score (MOS), which is an absolute category rating scale. The level of listening quality scale level was obtained with 5-stage absolute qualities: MOS 5.0- Excellent, MOS 4.0-Good, MOS 3.0-Fair, MOS 2.0- Poor, MOS 1.0-Bad. Eight younger Chinese-speaking listeners (6 females and 2 males) with the mean age of 24 were invited for evaluating the naturalness of the speech. They were presented sentences with flattened intonation, typical intonation, and exaggerated intonation separately. After listening to the speech sentences, one of the levels was selected as this participant’s evaluation of the naturalness of the speech. The average score of all the participants was the MOS score of the measured speech naturalness. Results showed that the MOSs of the speech naturalness of the sentences with typical intonation was 3.75, flattened intonation was 3.28125 and exaggerated intonation was 3.0 respectively. Thus, although there is still some tiny gap with the corpus of natural speech materials, the level of naturalness of the speech is satisfactory.

#### Masker stimuli

Each target sentence was combined with a masker. There are two types of masker used in the present study, speech masker and noise masker. The speech masker consisted of same type of Chinese “nonsense” sentences and was a loop of digitally combined continuous recordings of grammatically correct but semantically meaningless sentences (whose keywords did not appear in the target sentences) spoken by two other young female talkers. Three hundred common syllables were selected from the database published in the People’s Daily over one year, and 100 sentences published in People's Daily that including 300 common syllables, were selected as acoustic materials for producing speech masker. Each of the 50 speech sentences were spoken by two female talkers respectively was mixed using Matlab programming and a stream of steady-state Chinese speech masker with the duration of 10 s was obtained. The noise masker was steady speech spectrum [14].

#### Sound pressure level of speech stimuli

The sound level calibration was conducted using a Larson Davis Audiometer Calibration and Electroacoustic Testing System (AUDit and System 824, Larson Davis, Depew, NY) with a “slow”/“RMS” meter response. All the target sentences were presented at 60 dBA[22,23]. Masker levels were adjusted to produce the following four SNRs: -4, 0, 4, and 8 dB. The maker levels were equated for RMS amplitude, and SNR levels were computed in the same manner as in [24], by first removing pauses in the speech signal longer than 100 ms before calculating the RMS value.

### Design and procedure

In this study, each age group had three within-subject variables: (1) intonation condition (flattened, typical, and exaggerated intonation conditions), (2) masker type (speech masker and noise masker), and (3) SNR (four signal-to-masker ratios). Each of the 24 conditions contained 6 target sentences assigned to a single condition and one of the three voices in a random order.

A total of 144 trials were presented in 6 blocks with 1 block for each intonation condition under one masker type. The duration of each block was 15 minutes and comprised 24 trials. The blocks were separated by a short break to rest. The presentation order for the masker type/intonation-condition combinations was counterbalanced across participants, and the order of the four SNRs was arranged randomly for each masker type/ intonation-condition combination.

For a testing block, which was associated to a particular intonation type/ masker type combination, participants were firstly informed of the masker and intonation types before the formal testing. Within one block, under one particular intonation condition, the participant pressed the button of a response box to initiate the trial. Immediately after the button was pressed, a masker (i.e., a noise masker or speech masker) was gated on. Next, after a random period between 900 and 1000 ms after the masker onset, a target sentence started. Finally, the masker and target gated off simultaneously.

Participants were asked to loudly repeat the entire target sentence as best as they could immediately after all the stimuli ended in each trail. The experimenters, who sat outside the anechoic chamber, scored the number of correctly identified syllables for each keyword. To ensure all the participants adequately understood and correctly followed the experimental instructions, the experiment began with a training block (approximately 12 trials) to familiarize the participants with the task.

## Results

A logistic psychometric function,

$$y=1/\left[1+e^{-\sigma \left(x-\mu \right)}\right]$$ (1)

was fitted to each participant’s data by using the Levenberg-Marquardt method [25], where *y* is the probability of correct identification of the final keywords in the target sentences, *x* is the SNR corresponding to *y*, *μ* is the SNR corresponding to 50% correct on the psychometric function, and *σ* determines the slope of the psychometric function.

**Fig 3:**
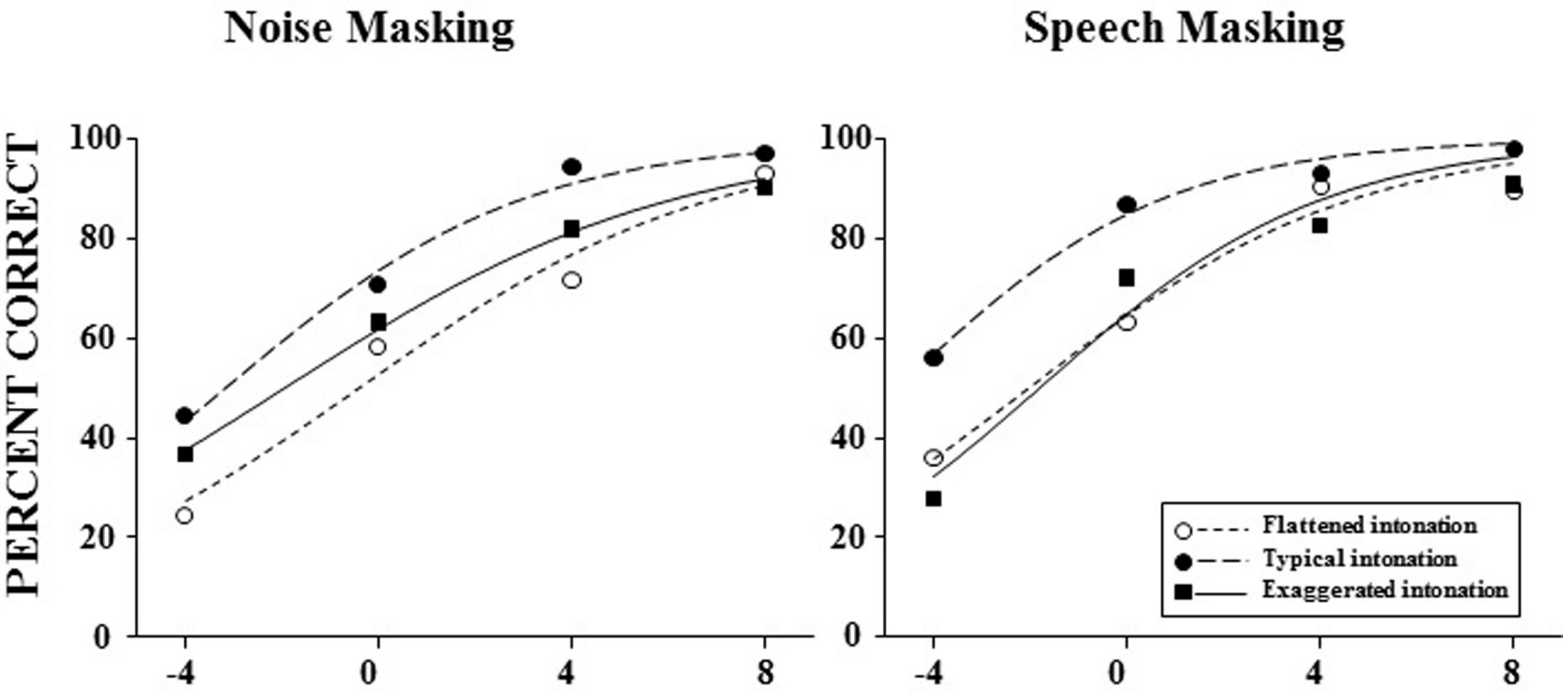
**Group-mean percent-correct syllable identification for the last target keyword as a function of the signal-to-masker ratio (SNR) for younger (top panels) and older (bottom panels) participants when the masker was noise (left panels) or speech (right panels) under each of the intonation conditions:** (1) flattened intonation conditions (open circles), (2) typical intonation conditions (filled circles), and (3) exaggerated intonation conditions (filled squares). Group-mean best-fitting psychometric function curves for the last keyword are shown in each of the panels.

Panels A and B in Fig 3 illustrate group-mean percent-correct word identification as a function of SNR along with the group-mean best-fitting psychometric functions (curves) for older (top panels) and younger (bottom panels) participants when the masker was noise (left panels) or speech (right panels) for the three intonation conditions: (1) flattened intonation (open circles), (2) typical intonation (filled circles), and (3) exaggerated intonation (filled squares).

**Fig 4:**
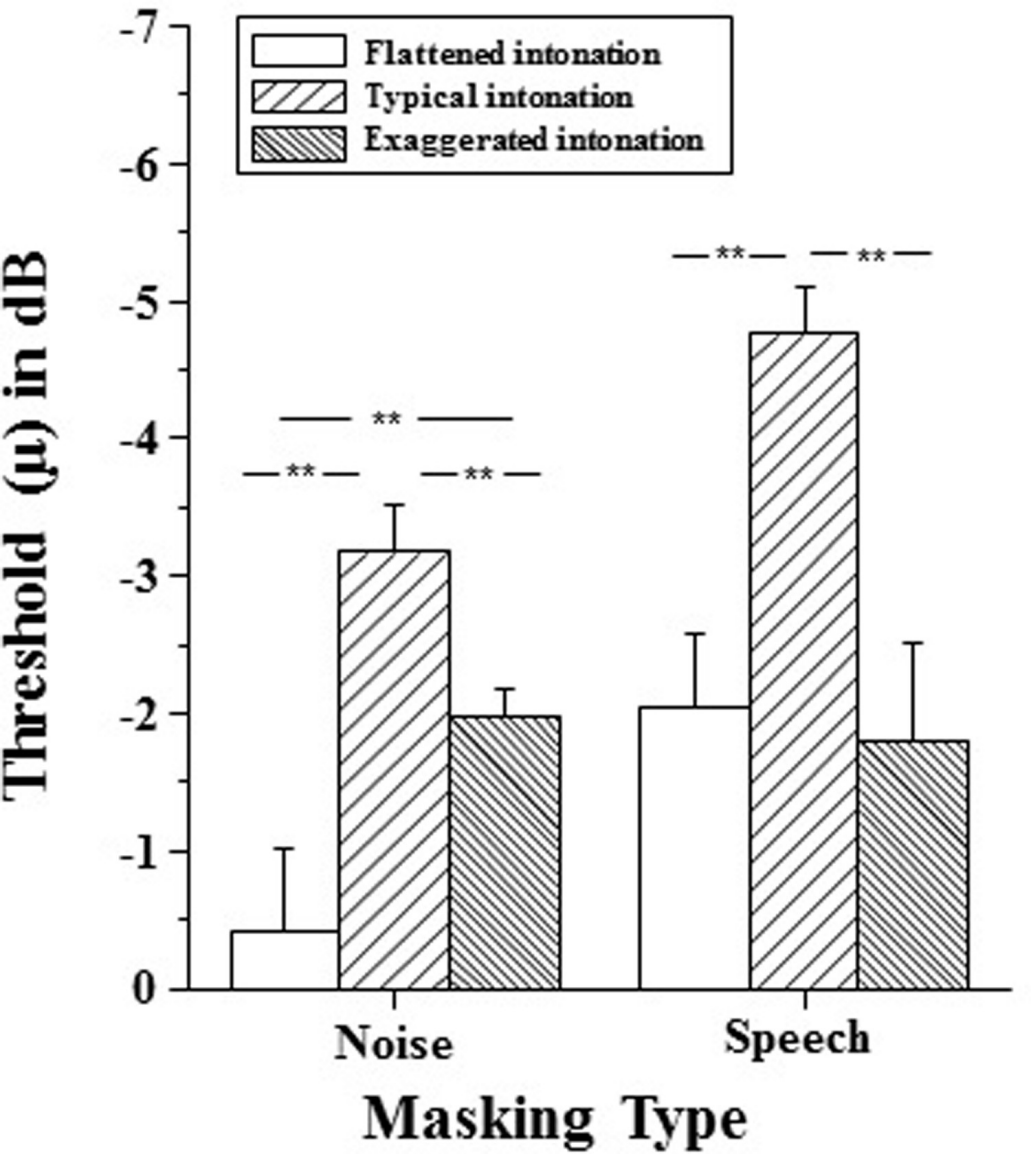
Group-mean threshold (*μ*) values for recognizing the final target keyword for each intonation condition when the masker was either noise or speech. Error bars indicate the standard errors of the mean. **: the difference was significant.

The differences in recognizing the keyword across intonation conditions were examined by analyzing the differences in 50%-correct thresholds (μ). Fig 4 plots averaged group-mean threshold (μ) values for recognizing the target keyword for each intonation condition when the masker was either noise or speech.

A 2(masker type)×3(intonation type) two-way within-subject ANOVA shows that the main effect of masker type was significant (F[1,11] = 12.000, *p* = 0.005),the main effect of intonation type was significant (F[2,22] = 12.000, *p* = 0.000), and the interaction between masker type and intonation type was significant (F[2,22] = 12.000, *p* = 0.000).

When the masker was noise, a one-way ANOVA showed that the intonation effect on μ was significant (F[2,22] = 12.000,p = 0.000), suggesting that presenting an individual sentence with different intonation affects recognition of the final keyword under the noise masking condition. Post hoc analyses with the adjusted α of 0.05 showed that the threshold under the typical intonation condition was significantly different from that under the exaggerated intonation condition (*p* = 0.016) and that under the flattened intonation condition (*p* = 0.016), and there is a significant difference in μ between the flattened and exaggerated intonation conditions (*p* = 0.013). Results revealed that intelligibility was reduced by the lack of natural intonation contours when in presence of background noise.

When the masker was speech, a one-way ANOVA showed that the intonation effect on μ was significant (F[2,22] = 7.463, *p* = 0.003), suggesting that the available of intonation cue affect the keyword recognition under the interference speech masking condition. Post hoc analyses with the adjusted α of 0.05 showed that the threshold under the typical intonation condition was significantly different from that under the exaggerated intonation condition (*p* = 0.039) and that under the flattened intonation condition (*p* = 0.046), however, the threshold under the flattened intonation condition was not significantly different from that under the exaggerated intonation condition (*p* = 1.000).

These results suggest that the difference in masking effect between the noise masker and the speech masker was significant. Moreover, the results indicated that the performance differs under the three intonation condition in the noise masker relative to their performance against a speech masker.

The results also indicated that although preserve tone information, speech intelligibility was reduced by the lack of natural intonation contours, whenever there is background noise or speech masker. Moreover, the results revealed that in both types of maskers, the performance under the exaggerated intonation condition or flattened intonation condition was much worse than the performance under typical intonation condition.

The results also showed that the performance under the exaggerated intonation condition in the presence of speech masker was much worse than the performance against a noise masker. The results revealed that intelligibility degraded to a greater extent for Chinese nonsense sentences with exaggerated intonation than that with flattened intonation when the types of masker changed from noise to speech.

## Discussion

### F0 contour contribute substantially to Chinese speech recognition in background noise

This study used different intonation cues to examine the speech unmasking effect of intonation perception. The results of this study supports earlier findings that any unnatural F0 contour manipulation decreased speech understanding in background noise [4,5]. This notion that the F0 contour is important in degraded speech understanding was also supported by the finding that, in this study, pilot listeners performed equally well across all three conditions when the sentences were presented in a quiet condition. When the stimuli were presented in noise, performance dropped. This finding is also in accord with the speculation proposed in the study by [26], who asserted that when the entire speech is degraded, listeners seem to rely more heavily on the F0 contour.

Specifically, the results of this study revealed that when presented in quiet, the speech perception of Chinese nonsense sentences with flattened F0 contours were as intelligible as those with natural F0 contours. By contrast, when presented in either noise or a speech masker condition, flattening the F0 contours of Chinese nonsense sentences dramatically reduced the intelligibility compared with the sentences with natural F0 contours. These results show a substantial contribution of F0 contour to sentence recognition under a noise environment.

The results in this study also highlighted the importance of natural F0 contours for sentence intelligibility, which were consistent with previous findings in [10,27].

### Unfamiliar intonation contour decreases releasing Chinese speech from masking

The results indicated that recognition of the final keywords was much better under the typical intonation condition than that under the flattened or exaggerated intonation condition, suggesting that an unfamiliar intonation contour slows releasing Chinese speech from masking. The results were consistent with [28], indicating that an unfamiliar intonation contour has a robust detrimental effect on the processing of speech.

Since all the redundant fine phonetic details including intonation are the lexical representation of words [29], an unfamiliar contour—to some extent—could result in some type of mismatch between the lexical representation and the perceived word, leading to the reduced word recognition.

According to our review of the literature, the information on intonation is processed and interpreted as soon as it becomes available[30]; thus, the increased difficulty in accessing the distorted intonation contour may result in bad performance for stimuli with an unfamiliar intonation contour.

The results also support Huang and Holt (2009) who assert that general auditory processing plays a role in lexical tone perception, that is, tone and intonation that share energy in the region of F0 but eliminate specific and speech-specific information should elicit similar effects on Mandarin tone perception[31].

Moreover, in Mandarin Chinese, lexical tones and intonation both depend on the variation of pitch. The speakers varied in F0 contour (i.e., how F0 changes during an utterance), and those differences in F0 contours between a target speaker and competing speakers can facilitate the tracking of the target voice when there are competing voices [32,33].

The results of this study showed that a flattened F0 contour within an individual sentence negatively influences the accuracy of speech recognition under the masking condition, regardless of the masker type. The results also indicated that the flattened fundamental frequency lowers releasing speech from masking, because it reduced the contrast between words and decreased the intelligibility of the utterance, which made it more difficult to parse continuous speech into meaningful units in a noisy environment.

Moreover, the results indicated that the intelligibility of speech under exaggerated intonation condition was worse than that under the flattened intonation condition whenever there is noise masker or speech masker, moreover, the exaggerated intonation suffers more in the speech-masker condition than that in the noise-masker condition.

### Except for the lexical tone contour, intonation information is also important for Chinese speech intelligibility

The results indicated that speech in a tone language (Mandarin Chinese) is harder to understand when the intonation is unnatural, even if you preserve tone information. That is, in addition to the lexical tone contour information, intonation information is also important for the Mandarin Chinese speech recognition.

Most of the research paradigms examining speech unmasking use task instructions that direct the listener to a semantic cue, often a key word within the target signal [34]. This study found that under a noise environment the participants also use indexical (tone in the voice, sentence intonation) cues to find the target utterance.

As in tonal languages, each monosyllable is pronounced with a distinctive tone that denotes a specific lexical meaning. In Mandarin Chinese, fundamental frequency serves as the primary acoustic cue for lexical tones and sentence intonation. Except for the lexical tone contour, sentence intonation is also important for the recognition of Mandarin sentences.

Patel et al,(2010) revealed that lexical prediction played an important role in helping the speech perception system compensate for lack of pitch variation, and top-down knowledge was helpful for enabling a listener to quickly realize the lexical intent of a spoken utterance and remap the vowel space [35,36]; thus, this study used non-semantic and meaningless Chinese nonsense utterances. One of the innovative features of this study was that each utterance was subjected to a re-synthesis technique that allowed flattening or exaggerating of the fundamental frequency while maintaining the timing and spectral characteristics of the utterances. In addition, different from English utterances, in Mandarin Chinese, the utterance of each syllable has its own pitch contour, and the pitch contour is more uniform across syllables. Notably, there seemed to be greater time-to-time variation in F0 in a Mandarin Chinese utterance than in an English utterance. Future studies could be conducted to help ascertain whether there is a difference between the Chinese and English utterances in the effective use of the intonation cues across the two languages.

The results of this study attempt to provide useful insights into the future development of speech communication applications, and the synthesis of intonation cues investigated in this study could be used to enhance the listener’s ability to extract information from target speech masked by simultaneous competing talkers in an interactive speech system.

## Conclusions

This study investigated the extent to which listeners can use intonation information to identify and selectively attend to a Chinese nonsense target utterance in noise and whether intonation cues affect the unmasking of Mandarin speech. The results of this study showed that recognition of the final keywords in the target sentence was much better under the typical intonation condition than under the flattened intonation condition or exaggerated intonation condition whenever there was a noise masker or a speech masker, suggesting that a flattened F0 contour or exaggerated F0 contour within an individual sentence negatively influences the accuracy of speech recognition under the noisy environment. Moreover, the results indicated that the performance under the flattened intonation condition was better than under the exaggerated intonation condition when there was a noise masker; however, the performance under the flattened intonation condition didn’t differ significantly from the exaggerated intonation condition when the masker was speech. These results revealed that listeners can take advantage of the troughs in the intonation of the Chinese speech masker to gain a “glimpse” of the Chinese target signal.
